# Gut microbiota disturbance during helminth infection: can it affect cognition and behaviour of children?

**DOI:** 10.1186/s12879-016-2146-2

**Published:** 2017-01-10

**Authors:** Vanina Guernier, Bradley Brennan, Laith Yakob, Gabriel Milinovich, Archie C. A. Clements, Ricardo J. Soares Magalhaes

**Affiliations:** 1 School of Veterinary Science, University of Queensland, Gatton, 4343 QLD Australia; 2 School of Public Health, University of Queensland, Herston, 4006 QLD Australia; 3 Princess Alexandra Hospital, Metro South Health and Hospital Services, Brisbane, Australia; 4 Department of Disease Control, London School of Hygiene and Tropical Medicine, London, UK; 5 Research School of Population Health, Australian National University, Canberra, Australia; 6 Children’s Health Research Centre, University of Queensland, South Brisbane, 4101 QLD Australia

**Keywords:** Central nervous system, Development, Gut microbiota, Helminths, Mental health, Microbiota-gut-brain axis

## Abstract

**Background:**

Bidirectional signalling between the brain and the gastrointestinal tract is regulated at neural, hormonal, and immunological levels. Recent studies have shown that helminth infections can alter the normal gut microbiota. Studies have also shown that the gut microbiota is instrumental in the normal development, maturation and function of the brain. The pathophysiological pathways by which helminth infections contribute to altered cognitive function remain poorly understood.

**Discussion:**

We put forward the hypothesis that gastrointestinal infections with parasitic worms, such as helminths, induce an imbalance of the gut-brain axis, which, in turn, can detrimentally manifest in brain development. Factors supporting this hypothesis are: 1) research focusing on intelligence and school performance in school-aged children has shown helminth infections to be associated with cognitive impairment, 2) disturbances in gut microbiota have been shown to be associated with important cognitive developmental effects, and 3) helminth infections have been shown to alter the gut microbiota structure. Evidence on the complex interactions between extrinsic (parasite) and intrinsic (host-derived) factors has been synthesised and discussed.

**Summary:**

While evidence in favour of the helminth-gut microbiota-central nervous system hypothesis is circumstantial, it would be unwise to rule it out as a possible mechanism by which gastrointestinal helminth infections induce childhood cognitive morbidity. Further empirical studies are necessary to test an indirect effect of helminth infections on the modulation of mood and behaviour through its effects on the gut microbiota.

## Background

Soil-transmitted helminth (STH) infections such as *Ascaris lumbricoides*, *Trichuris trichiura* and hookworms affect more than a third of the world’s population, with the heaviest worm burdens occurring in non-industrialized countries [1]. STH,infections are associated with significant morbidity, particularly in children; infection is recognised to cause nutritional deficits, with clinical and physical consequences including anaemia and reduced growth [2–5]. In addition to their nutritional effects, soil-transmitted helminth infections are also reported to impair cognitive function [6–8], limit educational advancement, and as a result, hinder economic development [9]. The effects of helminth infections, particularly by hookworms, on cognitive development of children were first reported by Waite and Neilson in 1919 [10]. Since then, there have been several studies that looked at the effects of STH infections on different domains of cognitive function. Nevertheless, at present, available evidence is conflicting. A recent update of the Cochrane review concluded that there is little or no evidence that cognitive function is affected by STH infections [11, 12], but some have argued that trials included in the Cochrane review were of poor quality to measure effects [13] and that STH effects on cognition could even be greater than initially suggested [3]. Several observational studies (some not included in the meta-analysis by the Cochrane review) reviewed in [13] reported that STH infections impair the efficiency of cognitive processes including memory, learning, verbal fluency and non-verbal intelligence [6–8, 14–17]. In addition, investigations into STH infections during pregnancy have also demonstrated an association with poor cognitive and motor development in infants [18, 19].

Multiple mechanisms have been demonstrated to explain STH-induced nutritional impairment in infected individuals: a loss of iron and protein through feeding on host tissues [20], an increasing malabsorption of nutrients [5], a competition for vitamin A in the intestine [21], or diarrhoea and dysentery [22]. Conversely, the pathophysiological pathways by which STH infections contribute to altered intellectual performance reported by some studies are still subject to significant debate. Some of the cognitive effects of STH infection can be partly explained by the direct effect of observed nutritional deficits on the brain and the indirect effect of pathophysiological events occurring in the gut environment where STH reside; the relative importance of these competing mechanisms remain unclear.

## Main text

### Interaction between gut microbial communities and the brain

There is a bidirectional functional communication between gut microbiota, the gastrointestinal (GI) tract and the central nervous system (CNS); these relationships have been recognised as the microbiota-gut-brain axis, which has been subject to substantial scientific enquiry in recent years [23–26]. The microbiota-gut-brain axis operates through a variety of physiological mechanisms, including neural, hormonal and immunological pathways [24].

Recent reports pointed to a crucial role of the microbiota-gut-brain axis in normal development, maturation and function of the brain [27–29], supported by emerging evidence that the disruption of the gut microbial community (i.e. dysbiosis) can affect emotional behaviour and related brain systems, which can lead to a range of abnormal or altered phenotypes, i.e. human brain diseases including autism spectrum disorder [30], anxiety, depression, and chronic pain [31, 32]. Clinical findings of these studies are supported by evidence primarily collected from experimental studies in rodents using various strategies: experiments using germ-free animals, experimental modification of the gut microbiota via antibiotics (downregulation) or prebiotics/probiotics (upregulation), or experimental infection with various pathogenic bacteria. Experimental studies on mice have shown how the microbial content of the GI tract influences feeding behaviours [33], stress-related behaviours [23, 34–36], pain perception pathways [37], and memory/learning development [36, 38].

### Gut microbiota and brain development

Mammalian brain development is initiated in utero, with rapid changes in neuronal organization [29]. But a considerable amount of morphological development, cell differentiation and acquisition of function takes place during postnatal development, with a striking increase in brain growth during the first 2 years of life [39]. Internal and external environmental signals such as nutrition, infection, the environment, or stress (maternal or environmental) can affect brain development until adulthood. As an example of diet impact, prolonged and exclusive breastfeeding has been shown to improve children’s cognitive development [40, 41].

Interestingly, the maturation of the gut microbiota occurs during the first 2 to 3 years of postnatal life, coinciding with a critical window of early brain development [42]. Initial colonization of the GI tract is dictated by the mother’s microbes (during the delivery) and the hospital environment, and further influenced by a number of factors including antibiotic use, diet, mode of delivery, environmental factors, or the maternal microbiota [43–45]. The gut microbiota plays a fundamental role in key systems regulating CNS development, especially synaptogenesis and myelination [39, 46]; this entails that a sustained imbalance within the microbial ecosystem of the infant gut could impair the cognitive development in early life. Insufficient or disturbed colonization of an infant resulting from Caesarean section and/or inadequate nursing could thus have unexpected outcomes.

### Interactions between helminths, gut microbiota and the host

One environmental factor impacting the gut microbiota is parasitic infection. The qualitative and quantitative alterations on the composition of the gut microbiota of the host upon helminth infection, and the underlying mechanisms leading to these changes have mainly been studied in animal models, specifically *Heligmosomoides polygyrus bakeri* infection in mice and *Trichuris suis* infection in pigs (reviewed in [47]). Such evidence in humans [48] or wild animals [49] under natural settings is scarce but also rarely investigated. A recent study on a population of wild mice naturally infected showed a modification in the diversity and composition of the gut microbiota, with evidence that the abundance of gut microbial taxa varies according to the helminth species colonizing the host [49].

A potential mechanism by which helminth infection could alter the gut microbiota composition is its effect upon the host immune system, which could disrupt the homeostatic relationship established between the gut microbiota and the host. Helminth effects on the host immune system can come about by direct competition for niche space in the GI tract, or primarily through host immunomodulation. The direct helminth-induced host immune responses include down-regulation of inflammation, mainly via a protective Th2 response [50, 51]. These changes have resistance and tolerance roles, establishing an environment that promotes parasite survival and a prolonged reproductive phase [50–53]. Helminth interaction with the host immune cells/molecules can also occur indirectly through the excretory/secretory products released by live worms [54]. These proteins with immunomodulation properties are involved in creating an anti-inflammatory (e.g. by induction of Th2 response) and immuno-tolerant environment (e.g. via host dendritic cells modulation) [55–57] that promote both helminth and host survival [51]. The host-helminth-microbiota interaction is thus a complex and dynamic relationship, and all three components must be considered to better understand helminth pathogenesis.

### Testing the helminth-gut microbiota-CNS hypothesis

**Fig. 1:**
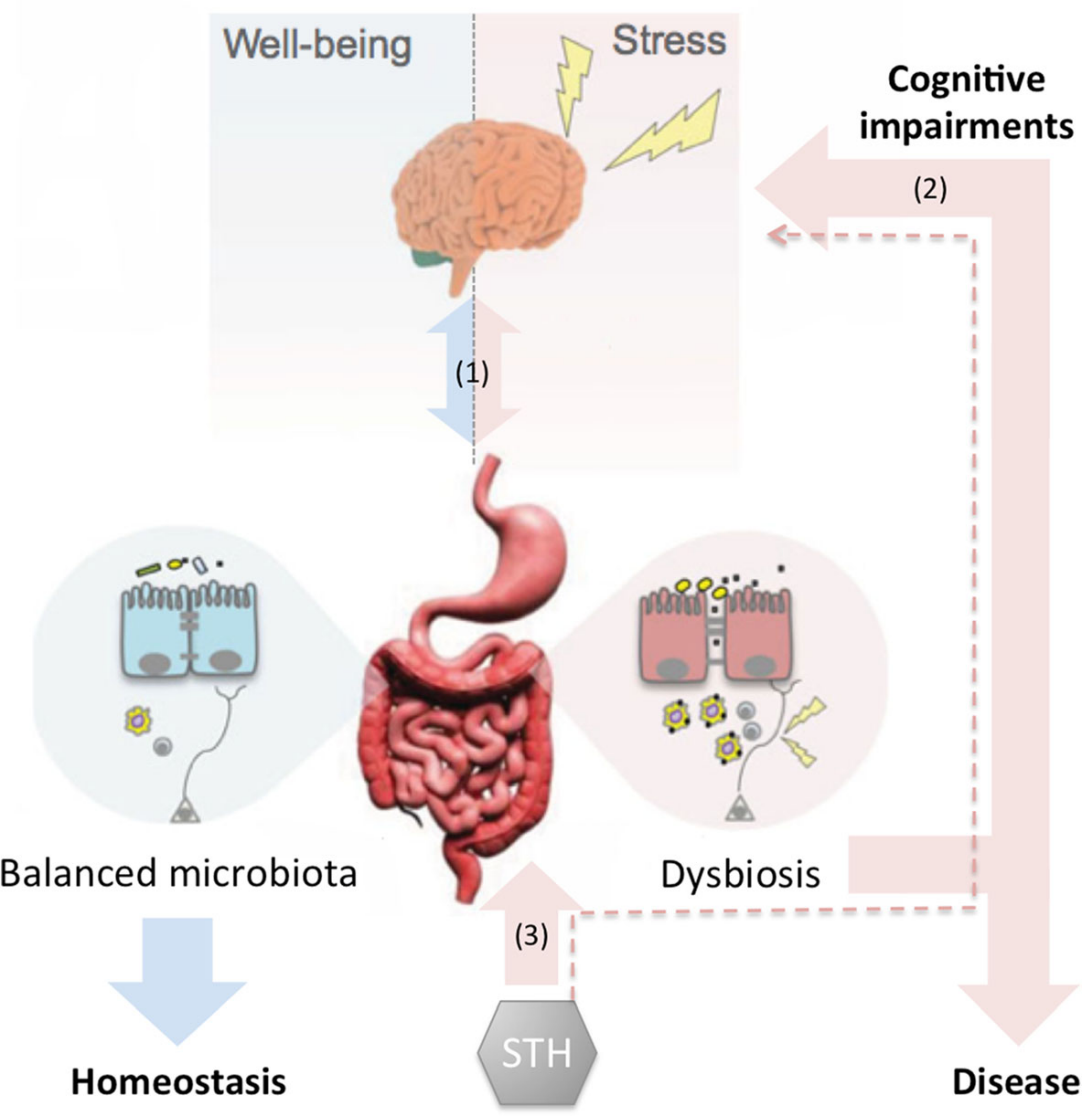
The microbiota-gut-brain axis and its interactions with soil-transmitted helminths (STH): (1) shows the bidirectional communication between the gut and the brain, which occurs through multiple pathways that include hormonal, neural and immune mediators; (2) shows the impact of gut microbiota dysbiosis on cognition; (3) shows the impact of helminth infection on the gut microbiota. The dotted arrow shows the hypothesized pathway leading from STH infection to cognitive impairments, potentially through its impact on the gut microbiota (i.e. dysbiosis). Adapted from De Palma et al. 2014 [65]

Available data suggests that helminths, the gut microbiota and the host should be viewed as a dynamic and integrated system [52]. In support of that view is current evidence pointing out that (1) helminth infection in infants is associated with significant cognitive impairment; (2) studies focusing on the gut-microbiota-brain axis demonstrate that the gut microbiota has a key role in early brain development and dysbiosis in the gut microbiota adversely affects cognitive abilities; and (3) STH infections result in gut microbiota dysbiosis. This suggests that any disturbance in homeostasis of the healthy gut microbiota, e.g. as a result of STH infection, is likely to impact on the host’s health (Fig. 1). We thus hypothesize that the effects on cognitive function associated with helminth infection can be partly explained by a secondary/indirect effect of the gut microbiota dysbiosis induced by infection.

While there is evidence on the interactions between different players in this complex system, the quantitative rates by which the players interact with each other cannot be informed from current literature. The helminth-gut microbiota-CNS hypothesis needs support from further experimental and epidemiological studies. Some reliable measurement tools exist to assess anxiety behaviours, working memory or pain perception, and they have been used in some studies investigating the impact of helminth-microbiota induced changes on those parameters. The specific micro-organisms and components involved in this process, the clinical relevance and the mechanisms underlying possible alterations of the microbiota composition in helminth-infected children require further population-based studies. These investigations would bring a novel and refreshing approach to scientific enquiry into the role of STH on cognitive function of children, with important implications for clinical practice by offering a range of potential therapeutic opportunities to target CNS developmental and behavioural disorders.

There is strong evidence linking parasite infections, particularly hookworms, to anemia [58], and many studies which link anemia to disturbances in social, emotional, and cognitive development (e.g. [59, 60]). Thus investigations could be extended to examine the role of parasite infection on cognitive impairment as an indirect result of anemia. Furthermore, recent studies showed that, unlike gut microbiota from healthy children, microbiota from undernourished children is immature [61–64] and can transmit growth impairment, as shown by faecal transplant experiments [61]. So it would be worth testing the potential impact of helminth infection on children growth via infection-induced microbiota dysbiosis through a similar loop analysis.

### Summary

We put forward the hypothesis that changes in the gut microbiota induced by helminth infections play an important role in cognitive morbidity of children (the helminth-gut microbiome-CNS axis). Factors supporting this hypothesis are: 1) the role that gut microbiota has on cognitive development; 2) the ability of helminth infections to change gut microbiota composition and diversity; and 3) the observed effect of helminth infection on cognitive development indicators. The hypothesis should be further tested using experimental and epidemiological studies.

## Conclusions

While available evidence in favour of the helminth-microbiota-CNS hypothesis is circumstantial, the recent debate around helminth associated morbidity indicate the need for further research to elucidate the mechanisms through which gastrointestinal helminth infections induce cognitive developmental morbidity. Future studies looking at the effect on STH on childhood cognitive developmental domains should be adequately powered to measure effects that are likely to be subtle. In addition future studies should considered validated tools for measuring cognitive morbidity effects; these need to be sensitive enough to detect quantitative changes in the microbiota and longitudinal study designs will be paramount to quantify the effects on each element of the system. Combining metagenomic output with comprehensive psychometric tests constitutes an important direction for future work.

## Abbreviations

CNS: Central nervous system
GI: Gastrointestinal
STH: Soil-transmitted helmiths
